# Inconsistent use of male condoms among HIV-negative men who have sex with other men

**DOI:** 10.1590/1518-8345.6327.3891

**Published:** 2023-04-28

**Authors:** Laelson Rochelle Milanês Sousa, Henrique Ciabotti Elias, Juliano de Souza Caliari, Aliete Cunha de Oliveira, Elucir Gir, Renata Karina Reis

**Affiliations:** 1 Universidade de São Paulo, Escola de Enfermagem de Ribeirão Preto, Centro Colaborador de la OPS/OMS para el Desarrollo de la Investigación en Enfermería, Ribeirão Preto, SP, Brasil.; 2 Becario de la Coordenação de Aperfeiçoamento de Pessoal de Nível Superior (CAPES), Brasil.; 3 Instituto Federal de Educação, Ciência e Tecnologia do Sul de Minas, Campus Passos, Passos, MG, Brasil.; 4 Escola Superior de Enfermagem de Coimbra, Unidade de Investigação em Ciências da Saúde: Enfermagem, Coimbra, Portugal.; 5 Universidade de Coimbra, Centro de Estudos Interdisciplinares, Coimbra, Portugal.

**Keywords:** Men Who Have Sex With Men, HIV, Sexual and Gender Minorities, Condoms, Sexual Behavior, Prevention and Control, Hombres que Tienen Sexo con Hombres, VIH, Minorías Sexuales y de Género, Preservativos, Conducta Sexual, Prevención y Control, Homens que Fazem Sexo com Homens, HIV, Minorias Sexuais e de Gênero, Preservativos, Comportamento Sexual, Prevenção e Controle

## Abstract

**Objective:**

to analyze the factors associated with inconsistent use of male condoms among HIV-negative men who have sex with other men.

**Method:**

a cross-sectional, analytical and nationwide study conducted online in all the Brazilian regions in 2020, via networks and in dating websites. Inconsistent condom use was defined as occasional use or as never using it. Descriptive statistical analyses were performed, as well as association and binary logistic regression tests.

**Results:**

inconsistent condom use was reported by 1,222 (85%) of all 1,438 participants. The “homosexuals” (ORAdj: 2.03; 95% CI: 1.14-3.59; *p*=0.016), “having a fixed partner” (ORAdj: 2.19; 95% CI: 1.55-3.09; *p*<0.001), “oral sex” (ORAdj: 2.41; 95% CI: 1.31-4.43; p=0.005), “insertive anal” (ORAdj: 1.98; 95% CI: 1.10-3.58; *p*=0.023) and “STI diagnosis” (ORAdj: 1.59; 95% CI: 1.13-2.24; *p*=0.007) variables were independently associated with inconsistent use of male condoms. The “receiving advice on HIV test from a friend” (ORAdj: 0.71; 95% CI: 0.52-0.96; *p*=0.028) and “sex worker” (ORAdj: 0.26; 95% CI: 0.11-0.60; *p*=0.002) variables were protective factors.

**Conclusion:**

the variables under study pointed to a strong relationship between steady partners and increased trust and low adherence to condom use, corroborating other studies.

## Abstract (es)

**Objetivo:**

analizar los factores asociados con el uso inconsistente del preservativo masculino en hombres VIH negativos que tienen sexo con hombres.

**Método:**

estudio transversal, analítico, nacional realizado de forma *online* en todas las regiones de Brasil, en 2020, a través de redes sociales y sitios de citas. El uso inconsistente del preservativo se definió como el uso ocasional o no usarlo nunca. Se realizaron análisis estadísticos descriptivos, pruebas de asociación y regresión logística binaria.

**Resultados:**

1222 (85%) de los 1438 participantes informaron uso inconsistente del preservativo. Las variables “homosexuales” (ORA: 2,03; IC 95%: 1,14-3,59; *p* = 0,016), “tener pareja estable” (ORA: 2,19; IC 95%: 1,55-3,09; *p*<0,001), “sexo oral” (ORA: 2,41; IC 95%: 1,31-4,43; *p* = 0,005), “anal insertivo” (ORA: 1,98; IC 95%: 1,10-3,58; *p* = 0,023) y “diagnóstico de ITS” (ORA: 1,59; IC 95%: 1,13-2,24; *p* = 0,007) se asociaron de forma independiente con el uso inconsistente del preservativo masculino. Las variables “recibió consejo de un amigo sobre la prueba del VIH” (ORA: 0,71; IC 95%: 0,52-0,96; *p* = 0,028) y “trabajador sexual” (ORA: 0,26; IC 95%: 0,11-0,60; *p* = 0,002) fueron factores protectores.

**Conclusión:**

las variables estudiadas indicaron que hay una fuerte relación entre las parejas estables y el aumento de la confianza y la baja adherencia al uso del preservativo, lo que coincide con otros estudios.

## Abstract (pt)

**Objetivo:**

analisar os fatores associados ao uso inconsistente do preservativo masculino entre homens HIV negativos que fazem sexo com homens.

**Método:**

estudo transversal, analítico, de abrangência nacional realizado *on-line* em todas as regiões do Brasil, em 2020, por meio de redes sociais e em sites de relacionamento. O uso inconsistente do preservativo foi definido como uso ocasional ou nunca ter usado. Foram realizadas análises estatísticas descritivas, testes de associação e regressão logística binária.

**Resultados:**

o uso inconsistente do preservativo foi relatado por 1222 (85%) dos 1438 participantes. As variáveis “homossexuais” (ORA: 2,03; IC 95%: 1,14- 3,59; *p* = 0,016), “ter parceiro fixo” (ORA: 2,19; IC 95%: 1,55-3,09; *p*<0,001), “sexo oral” (ORA: 2,41; IC 95%: 1,31-4,43; p = 0,005), “anal insertivo” (ORA: 1,98; IC 95%: 1,10-3,58; *p* = 0,023) e “diagnóstico de IST” (ORA: 1,59; IC 95%: 1,13-2,24; *p* = 0,007) foram independentemente associadas ao uso inconsistente do preservativo masculino. As variáveis “recebeu aconselhamento de amigo sobre teste de HIV” (ORA: 0,71; IC 95%: 0,52-0,96; *p* = 0,028) e “profissional do sexo” (ORA: 0,26; IC 95%: 0,11-0,60; *p* = 0,002) foram fatores de proteção.

**Conclusão:**

as variáveis estudadas apontaram uma forte relação das parcerias fixas com o aumento da confiança e uma baixa adesão ao uso do preservativo, o que corrobora com outros estudos.

**Highlights:**

**(1)** Sexual orientation was associated with inconsistent condom use.

**(2)** There was a relationship between steady partners and low adherence to condom use.

**(3)** Prior STI diagnoses were associated with inconsistent condom use.

**(4)** There was low adherence to consistent condom use.

## Introduction

Inconsistent use of male condoms is understood as occasionally or never using them in sexual relations^(1)^ and is reported in the international literature as an important focus of prevention actions among Men who have Sex with other Men (MSMs), given the high risk of acquiring and transmitting the Human Immunodeficiency Virus (HIV) and other Sexually Transmitted Infections (STIs)^(2)^.

Despite the prevention efforts and scientific advances, particularly in recent decades, which have enabled increased availability and incorporation of prevention methods, MSMs are disproportionately affected by infection with HIV and other STIs^(3-4)^. Consequently, use of male condoms is still a protagonist in the context of preventing HIV and other STIs in this specific population group. In addition, inconsistent condom use promotes a search for other preventive methods such as Post- Exposure Prophylaxis (PEP) and Pre-Exposure Prophylaxis (PrEP). Intrinsic factors such as unplanned sexual practices and consumption of alcohol and other drugs, in addition to the relationship with the sex partner, delineate how the prevention methods are used^(5)^.

Advances in the prevention methods have added options to devise a broader prevention plan, which is known as combined prevention disclosed in campaigns promoted by the Brazilian Ministry of Health, which is the combination in the use of two or more methods^(6)^. In addition to that, these advances seek to comply with what has been established by UNAIDS (United Nations Joint Program on HIV/AIDS) known as the 90 - 90 - 90 Goal, of which Brazil is a signatory and has signed commitments to diagnose, offer treatment and promote viral suppression of 90% of the population by 2030^(7-8)^.

The risk to acquire HIV is the result of a combination of socio-structural, behavioral and biological factors. Despite the biomedical advances in terms of prevention, the number of new cases is increasing. The Latin American region presents profound and generalized inequalities that lead to social and structural barriers affecting access to health services, particularly among the key populations. The new infection cases in this region have increased by 21% and 44% among gay individuals and other MSMs since 2010^(7)^.

Brazil is a country with significant estimates regarding the epidemic by the HIV infection, with nearly 1.5 million AIDS cases notified up to the end of 2020^(1,9)^. The epidemic in the country is concentrated in key populations (sex workers and their clientele, gay people and other men who have sex with men, drug users and trans individuals)^(7)^ with HIV prevalence of 0.4% in the general population^(10)^ and of 18.4% among MSMs^(6)^. Consequently, MSMs are considered a priority for HIV prevention as a result of vulnerabilities for acquiring and transmitting the infection^(11)^.

MSMs represent a category of interest for research studies related to the prevention of HIV and other STIs and encompass an extremely wide range of sexual orientations and gender identities.

Sexual orientation and gender identity are considered as social determinants of health in the public policies. Sexual orientation refers to the ability for emotional, affective or sexual attraction to people of the same gender, different genders or more than one type of gender. Currently, the visibility guidelines, even by the LGBTQIAP+ (Lesbian, Gay, Bisexual, Transvestite, Transgender, Transsexual, Queer or non-binary, Intersex, Asexual, Pansexual, and +, which corresponds to other denominations not included in the acronym) acronym, are as follows: asexual (a person who does not feel any sexual attraction regardless of the sex/gender of the other), bisexual (a person who relates to both sexes/gender), gay (a cisgender or transgender male person who relates to other male people), heterosexual (a person who relates to other people of the gender opposite to their own), homosexual (a person who is attracted to people of the same sex/gender as their own), lesbian (a cis or trans person who relates to other cis or trans women), pansexual (people who develop attraction to other people’s gender identity or sexual orientation)^(12)^.

Gender identity is a particular internal experience corresponding or not to the sex attributed at a person’s birth, including self-perception of the body and gender expressions (way of dressing, behaviors, mannerisms, etc.). Some examples of gender identity are as follows: agender (a person who does not identify with or does not feel belonging to any gender), cisgender (people who are not transgender and identify in all respects with the gender assigned at birth), non-binary (people who consider male-female binarism as limiting), transgender (people who transition between genders), transsexual (a person who has a different gender identity than the sex assigned at birth) and queer (people whose sexual orientation is not exclusively heterosexual and who question binarism)^(12)^.

Inconsistent use of male condoms between sero-different sex partners is one of the aspects linked to the increase in the number of HIV cases. In addition, a study carried out in inland São Paulo with people living with HIV in a sero-different sexual partnership identified factors associated with sexual behavior with greater exposure to risk situations: having a lower schooling level, casual partners, using alcohol during sexual intercourse and not receiving guidance on prevention of HIV sexual transmission^(13)^.

In turn, among the MSM population, in addition to the aspects linked to information and substance use^(5)^, living with more diverse sexual networks contributes to risky sexual behaviors such as multiple sexual partners and unprotected sex^(5,14)^. Unprotected anal sex is the greatest risk factor for sexual HIV transmission among MSMs^(15)^. Studies conducted with MSMs in low-, middle- and high-income countries show high rates of unprotected anal sex^(16)^. However, risk exposure occurs when there is not a wider prevention plan with different methods, both biomedical and barrier.

When used consistently, male condoms offer very high protection against HIV and other STIs^(14)^. However, consistent use among MSMs is low and unsatisfactory for prevention^(17-18)^. Although a number of Brazilian studies have documented inconsistent use of male condoms, they were conducted with very specific population groups, not including MSMs in a more direct way^(1,19)^. Consequently, there is lack of studies with national samples about factors associated with inconsistent use of male condoms among Brazilian MSMs. Given the above, the objective of this article was to analyze the factors associated with inconsistent use of male condoms among HIV-negative men who have sex with other men.

## Method

### Study design

A cross-sectional, analytical and nationwide study conducted by means of online data collection in all the Brazilian regions, guided by the Strengthening the Reporting of Observational Studies in Epidemiology (STROBE) tool^(20)^.

### Data collection setting

Convenience sampling was used and data collection occurred by making the online questionnaire available in virtual environments such as Instagram, Facebook, Twitter, WhatsApp groups, Telegram and dating apps for MSMs. The questionnaire was hosted in SurveyMonkey. All the Brazilian regions were reached by means of this strategy.

### Period

The data were collected between April and May 2020.

### Population

Men who have sex with other men from all the Brazilian regions were eligible for the study. This group includes people with different sexual orientations and gender identities, which allows greater scope to understand the variables under analysis.

### Selection criteria

The participants should meet the established inclusion criteria to take part in the study: being aged 18 years or older, identifying themselves as males, having Brazilian nationality, having access to the Internet and having had sexual relations with another man at least once in their lives. The exclusion criteria was not answering the question about consistent condom use (Do you use male condoms in all your sexual relations? NOTE: Consider oral sex as a sexual relation.)

### Definition of the sample

The study was conducted in all the Brazilian regions by means of convenience sampling. In total, the online questionnaire was accessed 1,830 times and, after refining the database, a total of 1,438 MSMs were reached who met the inclusion criteria and answered the questionnaire in full.

### Study variables

Descriptive statistics (frequency) were used to analyze the following variables: sociodemographic characterization: years of study; gender; sexual orientation; steady sexual partner; alcohol use; smoking; use of other drugs; multiple partners; most frequent sexual practice; history of HIV testing and counseling; sources of information on HIV prevention; previous diagnosis of Sexually Transmitted Infection; being a sex worker and PrEP use.

The dependent variable was “inconsistent condom use” and was assessed in a dichotomous way (Yes/No). Inconsistent use was defined as the action of occasionally or never using condoms, in the form of an explanatory note in the upper part of the variable in the questionnaire^(1)^. The independent variables were as follows: “years of study” (<11 years and >11 years of study); “gender” (cisgender man; transgender man; intergender and non-binary); “sexual orientation” (homosexual; heterosexual; bisexual; pansexual; asexual and other); “steady sexual partner” (Yes/No); “alcohol use” (Yes/No); “use of other drugs” (Yes/No); “smoking” (Yes/No); “multiple partners” (Yes/No); “most frequent sexual practice” (oral; receptive anal; insertive anal; observer and other); “HIV test some time in life” (Yes/No); “advice about HIV test from a health professional” (Yes/No); “advice about HIV test from a friend” (Yes/No); “receiving free male condoms in the last 12 months” (Yes/No); “reading information about HIV prevention on the Internet in the last 12 months” (Yes/No); “reading information about HIV prevention in printed materials in the last 12 months” (Yes/No); “STI diagnosis” (Yes/No); “sex worker” (Yes/No) and “using PrEP” (Yes/No).

### Instruments used for data collection

The questionnaire containing the study variables was divided into three parts: the first one contained the research title, the invitation to participate, the inclusion criteria and the Informed Consent Form with the following options: “I read the terms and I agree to take part in the research” and “I read the terms and I do not agree to take part in the research”. The participants had access to the questionnaire if they selected the option agreeing to take part in the research and, if not, the invitation was closed and the SurveyMonkey platform directed access to a closing and thank you page. The second part of the questionnaire contained questions related to characterization of the sample and the third part was targeted to sexual behaviors.

### Data collection

Data collection took place by making the questionnaire available online in social networks and dating apps for MSMs. The internauts had the option to click on the link and be directed the questionnaire in the SurveyMonkey platform.

### Data treatment and analysis

Binary logistic regression analysis was used to assess the influence of the independent variables on inconsistent use of male condoms. For statistical significance, p<0.05 was considered for inclusion of the independent variables in the regression model, resorting to the “enter” model. In addition, the “bootstrapping” procedure was performed (considering 1,000 resamples from the existing sample; 95% Confidence Interval and Corrected and Accelerated Adjustment, CAA)^(21)^ to adjust the model. The odds ratios were calculated: Unadjusted Odds Ratio (ORUnad) and Adjusted Odds Ratio (ORAdj). A 95% Confidence Interval and a 5% (α=0.05) significance level were considered. All the analyses were performed in the *Statistical Package for the Social Sciences* (SPSS) software, version 20.0. **Ethical aspects**

The study was approved by the Research and Ethics Committee of the Ribeirão Preto Nursing School, University of São Paulo, under opinion number 3,172,445. All the participants agreed with the Informed Consent Form in the online modality, by selecting the “I read the terms and I agree to take part in the research” option. It is emphasized that all the ethical aspects inherent to research studies involving human beings were met.

## Results

The study participants were 1,438 MSMs: 915 (63.6%) were aged between 18 and 28 years old, 1,156 (80,4%) had more than 11 years of study, 1,263 (87.8%) were cisgender and 1,190 (82.8%) were homosexuals. Regarding use of male condoms, it was observed that 1,222 (85%) reported inconsistent use. Table 1 shows the relationship of the characterization variables corresponding to the men who have sex with other men with inconsistent use of male condoms. This is the initial association before performing the regression model, which showed that, in the association of the variables, “years of study”, “gender”, “sexual orientation”, “steady sex partner”, “frequent sexual practice”, “advice on HIV test from a friend”, “STI diagnosis” and “sex worker” were those that resulted in statistically significant values (p-value<0.05) in relation to inconsistent use of male condoms among MSMs.

**Table 1 -:** Characterization of the men who have sex with other men and relationship of the variables with inconsistent use of male condoms (N = 1,438). Brazil, 2020

Variables	Inconsistent condom use			
	Yes	No	Total	p*
	1222 (85%)	216 (15%)	1438 (100,0%)	
**Age group (Full years old)**				
18-28	769 (84,0)	146(16,0)	915 (100,0)	0,23
29-39	381 (87,4)	55 (12,6)	436 (100,0)	
40+	72 (82,8)	15 (17,2)	87 (100,0)	
**Years of study**				
< 11	225 (79,8)	57 (20,2)	282 (100,0)	**0,006**
> 11	997 (86,2)	159(13,8)	1156 (100,0)	
**Gender**				
Cisgender men	1094 (86,6)	169(13,4)	1263 (100,0)	**<0,001**
Non-binary	108 (73,5)	39 (26,5)	147 (100,0)	
Other	20 (71,4)	8 (28,6)	28 (100,0)	
**Sexual orientation**				
Homosexual	1031 (86,6)	159(13,4)	1190 (100,0)	**<0,001**
Bisexual	137 (80,1)	34 (19,9)	171 (100,0)	
Other	54 (70,1)	23 (29,9)	77 (100,0)	
**Steady (sex) partner**				
Yes	514 (90,8)	52(9,2)	566 (100,0)	**<0,001**
No	708 (81,2)	164(18,8)	872 (100,0)	
**Alcohol use**				
Yes	940 (85,9)	154(14,1)	1094 (100,0)	0,074
No	282 (82,0)	62 (18,0)	344 (100,0)	
**Smoking**				
Yes	227 (83,5)	45 (16,5)	272 (100,0)	0,435
No	995 (85,3)	171(14,7)	1166 (100,0)	
**Use of other drugs**				
Yes	336 (86,6)	52 (13,4)	388 (100,0)	0,296
No	886 (84,4)	164(15,6)	1050 (100,0)	
**Multiple partners**				
Yes	592 (83,6)	116(16,4)	708 (100,0)	0,154
No	630 (86,3)	100(13,7)	730 (100,0)	
**Most frequent sexual practice**				
Oral	320 (88,2)	43 (11,8)	363 (100,0)	**0,001**
Receptive anal	423 (82,8)	88 (17,2)	511 (100,0)	
Insertive anal	406 (87,5)	58 (12,5)	464 (100,0)	
Observer	8 (66,7)	4 (33,3)	12 (100,0)	
Other	65 (73,90)	23 (26,1)	88 (100,0)	
**HIV† test some time in life**				
Yes	981 (85,2)	171(14,8)	1152 (100,0)	0,706
No	241 (84,3)	45 (15,7)	286 (100,0)	
**Advice about HIV† test from a health professional**				
Yes	600 (83,4)	119(16,6)	719 (100,0)	0,104
No	622 (86,5)	97 (13,5)	719 (100,0)	
**Advice about HIV† test from a friend**				
Yes	572 (83,0)	117(17,0)	689 (100,0)	**0,046**
No	650 (86,8)	99 (13,2)	749 (100,0)	
**Receiving free male condoms in the last 12 months**				
Yes	738 (84,5)	135(15,5)	873 (100,0)	0,559
No	484 (85,7)	81 (14,3)	565 (100,0)	
**Reading information about HIV† prevention on the Internet in the last 12 months**				
Yes	1025 (85,1)	180(14,9)	1205 (100,0)	0,841
No	197 (84,5)	36 (15,5)	233 (100,0)	
**Reading information about prevention in printed materials in the last 12 months**				
Yes	630 (84,6)	115(15,4)	745 (100,0)	0,648
No	592 (85,4)	101(14,6)	693 (100,0)	
**Having already being diagnosed with some STI‡**				
Yes	459 (88,8)	58 (11,2)	517 (100,0)	**0,002**
No	763 (82,8)	158 (17,2)	921 (100,0)	
**Sex worker**				
Yes	14 (51,9)	13 (48,1)	27 (100,0)	**<0,001**
No	1208 (85,6)	203 (14,4)	1411 (100,0)	
**Using PrEP§**				
Yes	70 (86,4)	11 (13,6)	81 (100,0)	0,709
No	1152 (84,9)	205 (15,1)	1357 (100,0)	

* Chi-square;

† Human Immunodeficiency Virus;

‡ Sexually Transmitted Infection;

§ HIV Pre-Exposure Prophylaxis

Table 2 shows the results of the model adjusted according to the data regarding inconsistent use of male condoms among men who have with other men living in Brazil and possible associated factors. The “homosexual”, “having a steady partner”, “oral sex as the most frequent sexual practice”, “insertive anal as the most frequent sexual practice” and “previous STI diagnosis” variables were independently associated with inconsistent use of male condoms. The “sex worker” and “receiving advice about the HIV test from a friend” variables were protection factors against inconsistent use of male condoms among men who have sex with other men.

It was observed that men who declared themselves homosexual were 2.03 times more likely to make inconsistent use of male condoms when compared to those classified in the “other sexual orientation” category (ORAdj: 2.03; 95% CI: 1.14-3.59; *p*=0.016). The men with steady partners were 2.19 times more likely to indulge in inconsistent use of male condoms when compared to those with no steady partners (ORAdj: 2.19; 95% CI: 1.55-3.09; *p*<0.001). The men whose most frequent sexual practice was oral sex were 2.41 times more likely to indulge in inconsistent use of male condoms when compared to those with other sexual practices (ORAdj: 2.41; 95% CI: 1.31-4.43; *p*=0.005). The men whose most frequent sexual practice was insertive anal sex were 1.98 times more likely to indulge in inconsistent use of male condoms when compared to those with other sexual practices (ORAdj: 1.98; 95% CI: 1.10-3.58; *p*=0.023).

The men who had already been diagnosed with a Sexually Transmitted Infection were 1.59 times more likely to make inconsistent use of male condoms when compared to those without a previous STI diagnosis (ORAdj: 1.59; 95% CI: 1.13-2.24; *p*=0.007). Having already received advice on HIV tests from a friend resulted in 0.71 times fewer chances of making inconsistent use of male condoms when compared to “not” having received advice (ORAdj: 0.71; 0.52-0.96; *p*=0.028). Being a sex worker presented 0.26 times fewer chances when compared to not being a sex worker (ORAdj: 0.26; 95% CI: 0.11-0.60; *p*=0.002).

**Table 2 -:** Adjusted model of the factors associated with inconsistent use of male condoms among men who have sex with other men living in Brazil, 2020

Variables	Unadjusted Odds Ratio [95% IC*]	*p†*	Adjusted Odds Ratio [95% IC*]	*p†*
**Years of study**				
< 11	0,63 [0,45-0,88]	0,007	1,01 [0,70-1,47]	922
> 11	Ref. ‡	Ref. ‡	Ref. ‡	Ref. ‡
**Gender**				
Cisgender men	2,58 [1,12-5,97]	0,026	1,73 [0,72-4,15]	0,22
Non-binary	1,1 [0,45-2,71]	0,823	0,97 [0,38-2,48]	0,96
Other	Ref. ‡	Ref. ‡	Ref. ‡	Ref. ‡
**Sexual orientation**				
Homosexual	2,76 [1,64-4,62]	<0,001	2,03 [1,14-3,59]	**0,016**
Bisexual	1,71 [0,92-3,17]	0,086	1,53 [0,79-2,95]	0,206
Other	Ref. ‡	Ref. ‡	Ref. ‡	Ref. ‡
**Steady (sex) partner**				
Yes	2,29 [1,64-3,19]	<0,001	2,19 [1,55-3,09]	**<0,001**
No	Ref. ‡	Ref. ‡	Ref. ‡	Ref. ‡
**Most frequent sexual practice**				
Oral	2,63 [1,48-4,63]	<0,001	2,41 [1,31-4,43]	**0,005**
Receptive anal	1,70 [1,00-2,88]	0,049	1,51 [0,85-2,67]	0,152
Insertive anal	2,47 [1,43-4,29]	<0,001	1,98 [1,10-3,58]	**0,023**
Observer	0,70 [0,19-2,57]	0,6	1,03 [0,26-4,06]	0,965
Other	Ref. ‡	Ref. ‡	Ref. ‡	Ref. ‡
**Advice about HIV§ test from a friend**				
Yes	0,74 [0,55-0,99]	0,046	0,71 [0,52-0,96]	**0,028**
No	Ref. ‡	Ref. ‡	Ref. ‡	Ref. ‡
**Having already been diagnosed with some STI||**				
Yes	1,63 [1,18-2,26]	0,003	1,59 [1,13-2,24]	**0,007**
No	Ref. ‡	Ref. ‡	Ref. ‡	Ref. ‡
**Sex worker**				
Yes	0,18 [0,08-0,39]	<0,001	0,26 [0,11-0,60]	**0,002**
No	Ref. ‡	Ref. ‡	Ref. ‡	Ref. ‡

* Confidence Interval;

† Chi-square;

‡ Reference;

§ Human Immunodeficiency Virus;

|| Sexually Transmitted Infection

## Discussion

This study identified that the factors associated with inconsistent use of male condoms among men who have sex with other men in Brazil were as follows: being homosexual, having a steady partner, oral sex, insertive anal practice and STI diagnosis. Having received advice about the HIV test from a friend and being a sex worker were protective factors against inconsistent use.

Research studies on a preventive method in the population of men who have sex with other men are pertinent and justified by the high HIV transmissibility rates and the significant increase in new cases in recent years in this group. Approximately 51% of the HIV infection reports are from homosexual and bisexual men, according to data from the Brazilian Ministry of Health^(22)^. Male condoms are still a relevant strategy incorporated in a broader prevention plan and encourage research studies on risk management and pleasure promotion^(5,23)^, as the method remains included in the group of preventive strategies used, combined prevention^(24)^.

Sexual orientation also exerted an influence on condom use. Preliminary results of a study presented at the International Congress on Health whose objective was to evaluate sexual habits and orientation in Brazilian men identified that 52.47% of the homosexual men reported that they did not use condoms in all their sexual relations^(25)^. The high risk for inconsistent condom use among homosexuals is already pointed out in other studies as a result of the decrease in the perception of risk by HIV due to the improvement in quality of life and other factors, such as the technological advances that favor access to platforms with specific content with unprotected sex images for MSMs^(26)^. Cultural advances on the concept of homosexuality, in addition to enabling more social acceptance, allowed it to be more evident in the public sphere; therefore, people who assume homosexual sexual practices are considered potential targets of preventive measures^(27)^.

Regarding steady partners and inconsistent condom use, a number of studies have already brought about the perception of unprotected sex in heterosexual relationships as a promoter of romance, trust and support for stability of the couple^(25)^. In addition, male condoms interfere with penile erection and with the perception of sexual pleasure, and there are reports of allergies to their material^(28)^. In turn, among MSMs, a Chinese study conducted with 343 participants found that more than 50% of the sexual intercourses were without condoms. In the aforementioned study, it was also reported that trust and intimacy in the relationship with a steady partner were positively associated with having unprotected anal sex, representing important obstacles in the prevention of HIV infection^(16)^.

The results presented in the current study also indicated that the most frequent sexual practices associated with inconsistent condom use were oral sex and insertive anal sex, which corroborates the results of other studies^(16,26-28)^. In fact, low adherence to consistent use of male condoms is pointed out as one of the main practices of vulnerabilities among the gay population and is justified by discomfort, decreased pleasure, lower perception of contracting HIV and lack of knowledge^(29)^.

It was also found that having been diagnosed with an STI was a factor associated with inconsistent use of male condoms. Therefore, despite the benefits of condom use, the MSM population does not seem to associate consistent use with protection against other infections^(29)^.

In relation to sex workers, knowledge about HIV and AIDS is substantial, even with difficulties accessing health services and vulnerabilities negotiating use in sexual relations^(30)^. Male sex work is wide in terms of possibilities and condom use can be subjected to bargaining and changes in the prices of services, with consequent influences on health care^(31)^.

The data from this study indicate that low adherence to condom use is still a worrying factor. Data from other countries confirm low adherence: 72.9% used condoms in a Spanish study conducted with 405 MSMs. The European study also identified that absence of risk perception, high self-esteem and greater search for sensations during sex were factors associated with inconsistent use. In contrast, high levels of sexual assertiveness and self-efficacy were protective factors^(32)^.

Although in this study variables such as alcohol use, drug use and having accessed information in printed materials and on the Internet were not statistically significant, they variables pointed out in other studies as related to inconsistent condom use^(26)^. Therefore, they need to be interpreted carefully because they influence adherence to the prevention practices.

This study presented results referring to a specific group with regard to sexual orientations. Reported by most of the interviewees, homosexuality confers health professionals the responsibility of improving contact with MSMs, reducing the likely manifestations of possible sexual preconceptions. Targeted educational interventions encourage improvements in prevention according to the sexual behavior of a given population^(33)^. When analyzing the factors associated with inconsistent condom use by Brazilian MSMs, the need for health care directed to sexual health is evidenced. The probable lack of training in some regions can curb complete and equitable care anamnesis^(34)^. Training should be based on the health priorities of the country indicated by the results showed with the findings.

This study presented results from a sample of participants from all Brazilian regions that included a wide range of different sexual orientations and gender identities, all identified as MSMs. The results showed a high rate of inconsistent use of male condoms and factors associated with their use among Brazilian MSMs. Previous studies have been conducted with other groups in the key population, although not focusing on MSMs. Thus, all this information can assist in important strategic actions to improve prevention in this group.

Given the above, it is relevant to emphasize the need for actions directed to the inclusion of MSMs in combined prevention strategies, as isolated use of male condoms proved to be inconsistent. The results pointed out in this research contribute to the advance in the already produced knowledge with regard to the factors associated with inconsistent use of male condoms among HIV-negative men who have sex with other men, which can guide the formulation, implementation and consolidation of specific public policies capable of including men of different sexual and gender orientations for the prevention and control of HIV and other STIs.

The study had limitations related to recruitment of the participants, as the research was conducted online on social networks and dating websites aimed at the LGBTQI+ population in general, which may have excessively represented men who identified themselves as homosexuals. However, despite these aspects, the sample consisted of men from all Brazilian regions.

## Conclusion

The variable under study pointed to a strong relationship between steady partners and increased trust and low adherence to condom use, corroborating other studies conducted with MSMs. Although condoms are a form of protection, there is a perceived need to encourage MSMs to discuss the meanings of trust/intimacy of the relationship with regard to condom use.

Thus, it is necessary to implement strategies that develop communication skills, control of the emotions and potential conflicts that arise during the communication process that lead to difficulty in negotiation and the adoption of preventive strategies regarding reinfection by HIV and other sexually transmitted diseases, such as consistent use of male condoms.
